# An Updated Narrative Review on Ergometric Systems Applied to Date in Assessing Divers’ Fitness

**DOI:** 10.3390/healthcare9081044

**Published:** 2021-08-13

**Authors:** Sven Dreyer, Johannes Schneppendahl, Fabian Moeller, Andreas Koch, Thomas Muth, Jochen D. Schipke

**Affiliations:** 1 Hyperbaric Oxygen Therapy, University Hospital Düsseldorf, Moorenstrasse 5, 40225 Düsseldorf, Germany; sven.dreyer@med.uni-duesseldorf.de (S.D.); j.schneppendahl@gmx.de (J.S.); 2 Department of Exercise Physiology, Institute of Exercise Training and Sport Informatics, German Sport University Cologne, 50933 Cologne, Germany; f.moeller@dshs-koeln.de; 3 German Naval Medical Institute, Maritime Medicine, 24119 Kronshagen, Germany; koch@email.uni-kiel.de; 4 Institute of Occupational, Social and Environmental Medicine, Medical Faculty and University Hospital Düsseldorf, Heinrich-Heine-University, 40225 Düsseldorf, Germany; Thomas.Muth@uni-duesseldorf.de; 5 Forschungsgruppe Experimentelle Chirurgie, Universitäts-Klinikum Düsseldorf, Moorenstrasse 5, 40225 Düsseldorf, Germany

**Keywords:** scuba, diving, fitness, ergometry, exertion

## Abstract

Many recreational divers suffer medical conditions, potentially jeopardizing their safety. To scale down risks, medical examinations are mandatory and overwhelmingly performed using bicycle ergometry, which overlooks some important aspects of diving. Searching ergometric systems that better address the underwater environment, a systematic literature search was conducted using the keywords ‘diving’, ‘fitness’, ‘ergometry’, and ‘exertion’. All presented alternative systems found convincingly describe a greatly reduced underwater physical performance. Thus, if a diver’s workload in air should already be limited, he/she will suffer early from fatigue, risking a diving incident. How to assess fitness? Performance diagnostics in sports is always specific for a modality or movement. Therefore, professional scuba divers should be tested when fin-swimming underwater. For the vast number of recreational divers, the current screening can likely not be replaced. However, to prevent accidents, divers need to understand and be able to improve factors that limit their physical performance underwater. Other systems, presented here, will continue to be important tools in underwater research.

## 1. Introduction

Many recreational divers suffer medical conditions that potentially have an impact on their safety. In particular, cardiovascular conditions represent a hazard for scuba divers [1] as well as for breath-hold [2] divers (for review see: [3]). To prevent accidents, medical screening of divers for physical fitness is necessary. Here, bicycle ergometry in ambient air is the predominant screening method used [4,5].

However, compared to cycling in ambient pressure, important physiologic changes result during scuba diving. These include the use of a regulator for breathing, blood shifting into the thorax due to submersion, loss of body temperature due to convection, and the use of different muscle groups.

We conducted a systematic literature review using the keywords ‘diving’, ‘fitness’, ‘ergometry’, and ‘exertion’ searching PubMed, Google Scholar, Scopus, and Rubicon Archives.

At first, reasons for dive accidents and even fatalities will be presented shortly. Then, ergometer systems that were specifically developed to assess the diver’s physical fitness are presented. This mini review is completed with concluding remarks.

## 2. Accidents and Fatalities

The environmental factors in diving constitute numerous risks for scuba as well as for breath-hold divers. As a consequence, injuries and deaths are not uncommon in both types of divers [6]. However, there is no reliable information about incidences or relative risks neither for scuba divers nor for breath-hold divers [7]. According to an estimate, the individual risk for accidents during scuba diving is 1:6.000. This compares to a risk of 1:6.500 for motor vehicle accidents [8].

The most commonly reported triggers for accidents and fatalities include exertion, panic, buoyancy problems, disorientation and confusion [9]. As probable causes, insufficient training and pre-existing medical conditions were described [10,11]. To further underline that fitness to dive was a key point, one study reports on more than 80% of overweight divers (BMI > 25 kg/m^2^) in cases of fatalities [12].

Exertion during diving will lead to fatigue [13] that is defined as a disabling symptom in which physical function is limited [14]. Unfortunately, not only motor competence is diminished [15], but also cognitive performance [16,17], i.e., remembering, concentrating, and decision making will be impaired. It becomes obvious that exercise and physical capacity play a crucial role in preventing injuries and deaths [18].

To prevent accidents it has been proposed that all scuba diving candidates must successfully complete a medical examination to determine their medical, physical, and psychological fitness prior to their training [19]. The scope of this mini review is limited to physical fitness.

## 3. Ergometer Systems

### 3.1. General

Physical fitness is a requirement to meet the increased physical demands during diving. Reserves in both strength and aerobic capacity are important [20]. One measure to quantify physical fitness are metabolic equivalents of the task (MET). These dimensionless multiples of assumed resting metabolic rate range between 5 and 20 MET in the healthy population. the usual recreational dive requires a moderate energy expenditure and a 7-MET (~110 W) capacity seems generally adequate. However, an increased aerobic fitness is strongly recommended [21], and a minimum capacity as high as 13 MET (~200 W in bicycle ergometry) has been proposed for diving [20]. In addition, aerobic exercise training is associated with modest improvements in attention and processing speed, executive function, and memory, i.e., with neurocognitive performance [22].

To be regarded as fit to dive in Germany, 3 W/kg body mass at exhaustion are required for males on the bicycle ergometer. Hence, with a body mass of 75 kg, a young man needs to achieve roughly 225 W. Beginning at an age of 40 years, 10% per decade can be deduced, i.e., the same person at the age of 70 years, needs to achieve only roughly 100 W on the bicycle ergometer. However, do the physical demands implied by an underwater current respect the diver’s age?

In vast contrast, functional fitness testing of scuba divers is described to employ the following capabilities: (1) lift and carry individual items of diving equipment on land; (2) stand from sitting and walk 30 m in standard scuba equipment; (3) ascend a 1.5 m vertical ladder from the water wearing standard scuba equipment; and (4) swim underwater at 0.25 m s^−1^ for 30 min and at 0.6 m/s for 3 min wearing standard scuba gear [23].

### 3.2. Bicycle Ergometry

According to international guidelines, the physical fitness to dive is generally assessed using a bicycle stress test in ambient air [24,25]. This test has the advantage of not depending on particular skills, as almost all candidates know how to cycle. Unfortunately, due to the several differences between cycling and diving, the applicability of these results for the fitness to dive is reduced. Although large muscle groups are involved in biking, they differ significantly from muscle groups needed for fin-swimming (the calves, hamstring, quadriceps, and thighs). Unfortunately, the latter ones are frequently trained insufficiently which results in cramps during [26] and after [27] the dive. The emergence of cold-related cramps in the water is remembered.

Three variations from routine spiroergometry that is performed at an ambient pressure of 1 bar are outlined briefly. Usage of a breathing regulator during ergometry decreased maximum VO_2_ by roughly 15% compared to regular bicycle ergometry [28]. To account for pressure induced effects while diving, exercise was performed on a bicycle in a hyperbaric chamber at 1 bar and at 4 bar. During heavy exercise at 4 bar, ventilation, breathing rate and heart rate were significantly decreased compared to the results in ambient air [29] and therefore reduce maximum work rate capacity. Thirdly, immersion into the water shifts blood from the periphery into the thoracic cavity. As a result, maximum oxygen uptake, maximum pulmonary ventilation, and maximum heart rate were significantly lower in swimming compared to cycling [30].

There are a few under-water ergometers that aim to better assess a divers’ physical fitness. They all have been developed to overcome shortcomings of the traditional on-land bicycle ergometers to mimic the demands of an autonomous diver using fins for horizontal propulsion.

### 3.3. Underwater Bicycle Ergometry

As a first consequence, bicycles were placed under water [31]. Early results from under-water cycling candidates working against mild and moderate loads in warm water exhibited comparable responses to exercise in air [32]. Similarly, the in-air bicycle stress test correlated well with the cardio-circulatory performance, however underwater the gross capacity was decreased by about 50% compared to a given workload in air [24]. This shows, that cycling exercise is less efficient underwater than in air [33]. Likely, such differences could be due to an increased pedal drag and decreased pressure against pedals due to positive buoyancy [34].

Physical performance will further decrease with increasing depths due to the increasing ambient pressure leading to elevated energy costs of breathing. While one study makes increased airway resistance responsible for these costs [35], another study claims that increased breathing gas density does affect parameters such as submaximal or maximal VO_2_, VCO_2_, VE, or heart rate [36]. The latter notion is supported by a study looking at fresh water dives (20 m; 5 °C) in which the air has a higher density requiring a greater effort for breathing, i.e., to a parameter not taken at all into consideration by routine ergometry [37]. Not of surprise, respiratory muscle fatigue might become a limiting factor for underwater swimming performance [38], thus limiting motor competence and cognitive performance. Consequently, respiratory muscle training improves swimming endurance at depth [39] and reduces work of breathing [40,41] that, in turn, reduces breathing air consumption and helps reduce the nitrogen load.

Additionally, overlooked by routine ergometry, was the finding that comparable workloads lead to significantly higher blood pressures underwater compared to on-land [42]. Especially in candidates suffering high blood pressures, this further elevation of blood pressure would not be detected in routine screening.

Although many shortcomings of in-air bicycle ergometry could be overcome with the underwater method described above, the effort needed on a bike concerning the muscle groups used differs significantly from diving. To illustrate the consequences: screening tests of US Navy Fleet Divers had limited utility for physical selection as, for example, roughly one quarter of the candidates were unable to complete fin-kick tasks [43].

### 3.4. Stationary Fin Swimming

Other test systems have been developed to mimic diving characteristics more closely. Thus, the propulsive force can be assessed using a system that allows the diver to stay stationary in a horizontal position while fin-swimming and pushing against vertical handles connected to a load cell on a wall. In accordance with traditional ergometers, the power could be varied in steps via the kick frequency until VO_2max_ had been reached. The authors recommend their system for the assessment of the individual fin swimming capacity of candidates by measuring propulsive force and VO_2_ simultaneously and continuously [44].

Another system tested the performance of divers while swimming on the surface or during scuba diving in shallow water. Here, a tethered swim/counterweight system was used to provide graded exercise to exhaustion. In this setting, VE was not different, but VO_2max_ was significantly lower during diving compared to surface swimming [45].

In another complex system, a Buffalo-type hyperbaric chamber facility was employed [46]. Here, fully equipped divers were fixed in horizontal swimming position to a mechanism with suspended weights that pulled the diver back. Thus, to stay stationary, the diver needed to fin-swim with different intensities to compensate for the retraction force of different weights. This testing setup allows the investigation of various aspects of scuba diving, such as increased loads until exertion, breathing gas analysis, ambient pressures up to 6 bar, and various breathing gases. Using this testing setup, the superiority of the hip/thigh swimming style of well-trained divers over the knee/calf style of beginners could be demonstrated. In addition, efficiency of fin-swimming at all was surprisingly low with only 13% at low loads and fell to about 10% at high loads. Still, this low value compares well with earlier achieved data from scuba divers in a flow channel that varied between 1.2% and 5.6% [47]. For comparison: efficiency for cycling—as used by routine testing—amounts to 25% [48].

### 3.5. Real Fin Swimming

The three ergometer systems described above showed the capability to disclose reductions in physical fitness while diving, that cannot be verified by in-air bicycle ergometry; all setups had in common one shortcoming: investigating divers not moving through the water. Similar to candidates being evaluated while stationary on a treadmill, they were not confronted with the water resistance. Thus, ergometers were developed accounting for drag while moving in the water.

This drag depends on factors such as the swim velocity (=active drag) and the swimmer’s morphology and body position (=passive drag). In the case of scuba diving, passive drag also depends on the equipment and its arrangement. Unexpectedly, the location and density of the gear does alter the diver’s attitude in the water and increases the energy cost of swimming by 30% at slow speeds [49].

To further investigate drag effects, the VO_2_ was measured during swimming in a round pool with a 60 m circumference in thermoneutral water. In this setting, the mechanical efficiency amounted to 2.9% and 7.4% for a velocity of 0.5 m/s and 1.2 m/s, respectively [49], once more showing the rather low efficiency of fin swimming while scuba diving.

More recently, a similar fitness test (fit2dive) had been presented that also fulfils many requirements of an underwater ergometer. On a pool bottom a hexagonal parcourse with a 50 m circumference is marked [50]. Each of five equidistant points (10 m) is labelled. In addition to their scuba equipment, divers use a stopwatch and a marching table to correctly increase their swimming speed to perform graded workload until exhaustion. This test enables continuous assessment of heart rate and consumption of air and other breathing gases. Thus, post-dive analysis of the aerobic threshold and heart rate variability is possible, if equipped with an underwater Holter monitor. Moreover, a safety diver in the center of the hexagon can record videos to analyze the fin-swimming technique and equipment configuration.

However, one aspect of this testing setup needs to be discussed thoroughly: according to the literature, ergometer testing needs to create linear increases in heart rate, ventilation, and VO_2_ along with the increasing loads [51]. In concordance with an earlier study [52], data from the fit2dive test showed a curvilinear relation, because at increasing swim speeds the water resistance, i.e., the drag, increases with the square of the velocity.

Nevertheless, we suggest that the fit2dive test also be used to perform scientific studies. These could further extent the knowledge on topics such as the relation between exercise intensity and cognitive competences [53] or on the effects of oxygen-enriched air on physiological variables (Zenske et al., 2019).

Another testing setup described utilizes a scuba diver moving with different velocities through the open water holding the handles of a board in front of him. Between the handles and the board, a scaled, spring-balanced piston assembly permitted adherence to given fin-swimming velocities [54]. This system showed major differences in terms of VO_2_ and heart rate between rest and fin swimming at 0.4 m/s but almost no depth-dependent differences. In contrast, using this ergometer in a later study demonstrated that underwater VO_2max_ at 2 to 4 bar ranged from only 89% to 39% VO_2max_ observed on land [55].

## 4. Concluding Remarks

Professional divers in Germany have to undergo bicycle ergometry during prophylactic occupational medical examinations. The explanatory power of these tests is discussed controversially for the specific workload of a diver’s occupation [56]. In line, studies suggest that the current screening test has limited utility for physical selection purposes [43]. Here, a freely breathing, sitting candidate in thermoneutral air pedaling is utilized to mimic a diver breathing through a regulator and fin-kicking in a horizontal position to overcome the water resistance. This contrast seems to further justify the criticism. Additionally, not even taken into account is the greater work of breathing air of higher density and also the low temperatures, i.e., two parameters not taken into consideration by routine ergometry [37]. In the cold, maximal cardiac output is reduced and skin and muscles are vasoconstricted, resulting in a further reduction in exercise capacity [57]. Thus, if the physiologic capacity of a diver is already limited when tested in air, such a diver will suffer early from fatigue in a situation causing stress underwater. Reduced functional reserve will be associated with an increased risk of a diving accident [58].

Because of the reduced functional reserve, the problem of older divers—an increasing percentage—is shortly addressed. It has been suggested to distinguish between the chronological and the physiological age and, thus, make decisions about participation in scuba diving depending on fitness, comorbidities, and mobility/strength [59].

The search for a universal testing setup for all kinds of divers remains controversial. Whereas there is evidence that professional scuba divers, such as military divers or rescue divers should be examined using a system utilizing fin-swimming, these testing setups are rather extensive. Still, a more specific and in-water activity-related medical examination might also be desirable for recreational scuba divers [60]. Due to logistic issues, these setups may not be suitable for the vast number of recreational divers to replace current screening modalities. However, to prevent accidents, divers need to be comprehensively instructed about the factors that will limit their physical performance while scuba diving [61]. Additionally, the psychological fitness for survival in the subaquatic environment has not even been discussed.

Nevertheless, the evaluation with a cycle ergometer test is recommended in any case to reduce the risks of a sport in an extraordinary environment.
